# Supplementary material for: Metabotropic Glutamatergic Signaling Adaptively Controls Trans-thalamic Communication
Source: bioRxiv. 2026 Jul 28:2026.07.25.740691. Preprint. [Version 1] doi: 10.64898/2026.07.25.740691 (PMC13618427; doi:10.64898/2026.07.25.740691)
Supplement: Supplementary file 1 [file NIHPP2026.07.25.740691v1-supplement-1.pdf]

bioRxiv preprint doi: https://doi.org/10.64898/2026.07.25.740691; this version posted July 28, 2026. The copyright holder for this preprint (which was not certified by peer review) is the author/funder, who has granted bioRxiv a license to display the preprint in perpetuity. It is made available under a CC-BY 4.0 International license.

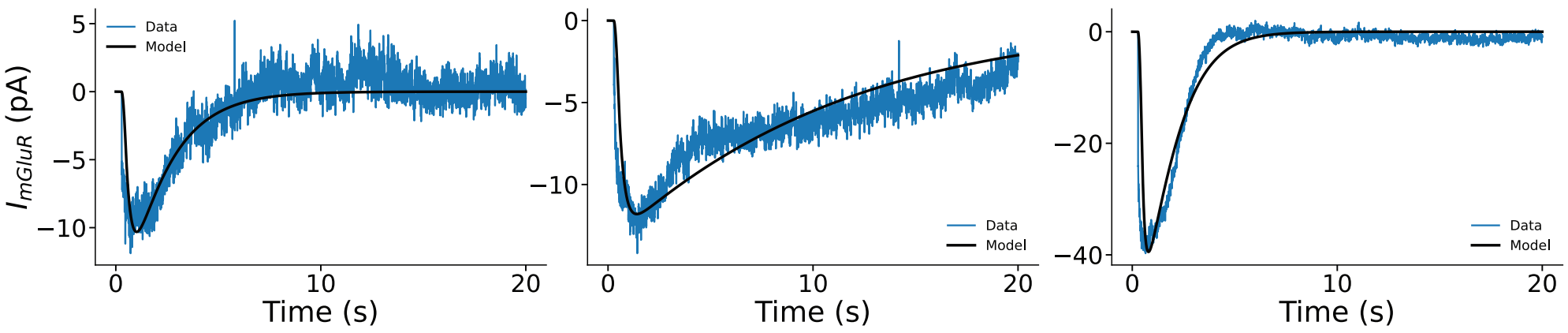


**Fig. S1 Additional example fits.** Three example mGluR EPSCs fit with $\alpha_{\mathrm{DAG}} = 0.0997$ $\mu$M, $\tau_{\mathrm{DAG}} = 1820$ ms (left) , $\alpha_{\mathrm{DAG}} = 0.09067$ $\mu$M, $\tau_{\mathrm{DAG}} = 10000$ ms (middle), and $\alpha_{\mathrm{DAG}} = 0.5711$ $\mu$M, $\tau_{\mathrm{DAG}} = 1166.03$ ms (right).

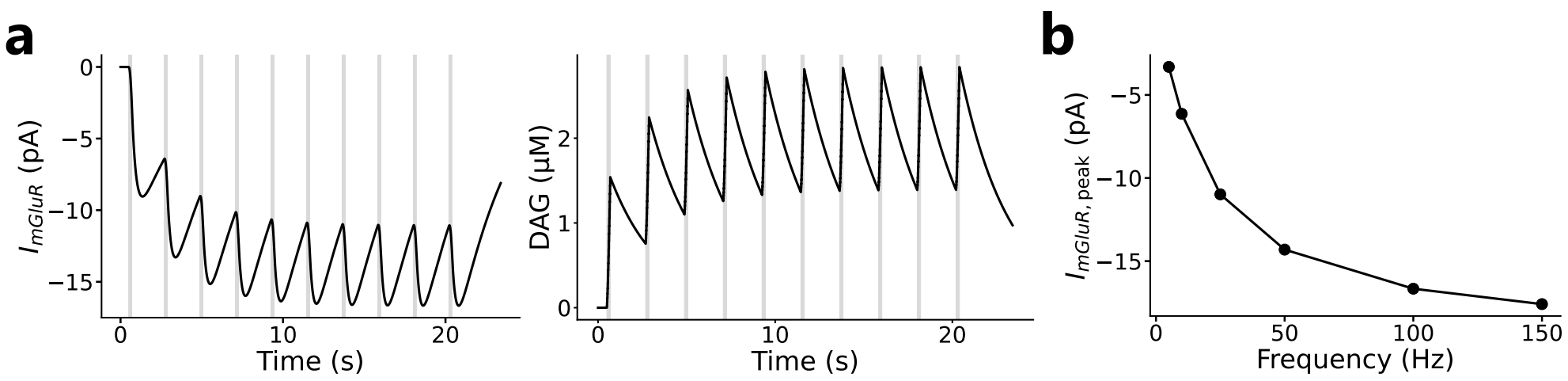


**Fig. S2 Slow Kinetics of mGluR in the presence of short-term synaptic facilitation** An example synapse onto model cerebellar unipolar brush cells is endowed with short-term synaptic facilitation dynamics. **(a.)** Even in the presence of facilitation, with $\alpha_{\mathrm{DAG}} = 0.135$ $\mu$M, $\tau_{\mathrm{DAG}} = 2800$ ms, the model displays accumulation of mGluR EPSCs resulting from presynaptic input ($20 \times 100$ Hz, time period denoted by gray bars) due to DAG's slow decay. **(b.)** The saturated peak mGluR EPSC as a function of input frequency has an equivalent relationship as what is seen in Fig. 2.

bioRxiv preprint doi: https://doi.org/10.64898/2026.07.25.740691; this version posted July 28, 2026. The copyright holder for this preprint (which was not certified by peer review) is the author/funder, who has granted bioRxiv a license to display the preprint in perpetuity. It is made available under a CC-BY 4.0 International license.

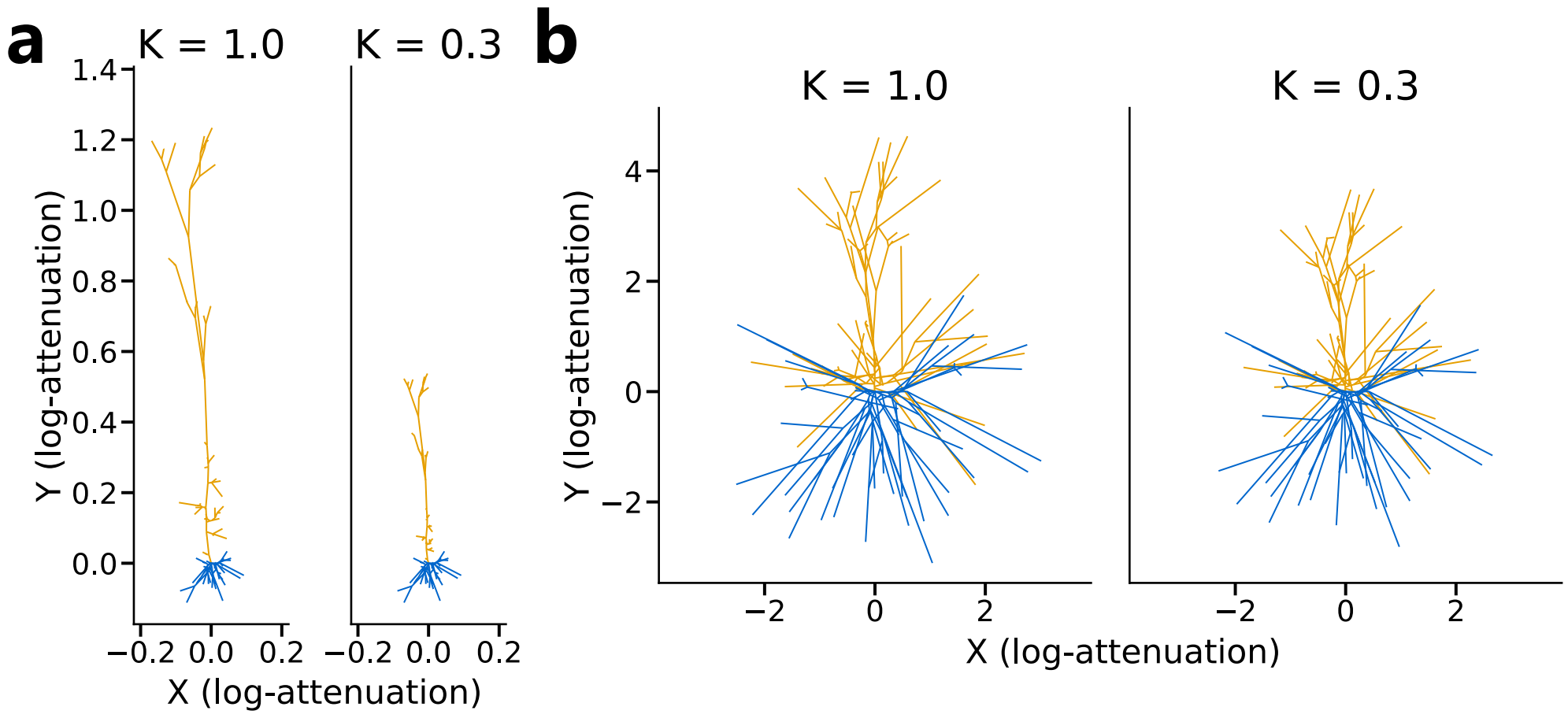


**Fig. S3 Functional attenograms of L5 PN following K2P suppression (a.)** Centrifugal somatic attenogram. Distance in this space corresponds to the log attenuation of voltage propagating from the soma toward distal dendrites (see Methods for details). From left to right, increasing mGluR-mediated suppression of K2Ps in apical dendritic compartments reduces attenuation, indicating that somatic depolarizations propagate more effectively into distal dendrites. **(b.)** Centripetal somatic attenogram. Distance in this space corresponds to the log attenuation of voltage propagating from the dendrites toward the soma. From left to right, increasing mGluR-mediated suppression of K2Ps in apical dendritic compartments reduces attenuation, reflecting stronger dendritic integration and electrotonic shortening of distal apical branches to the soma.

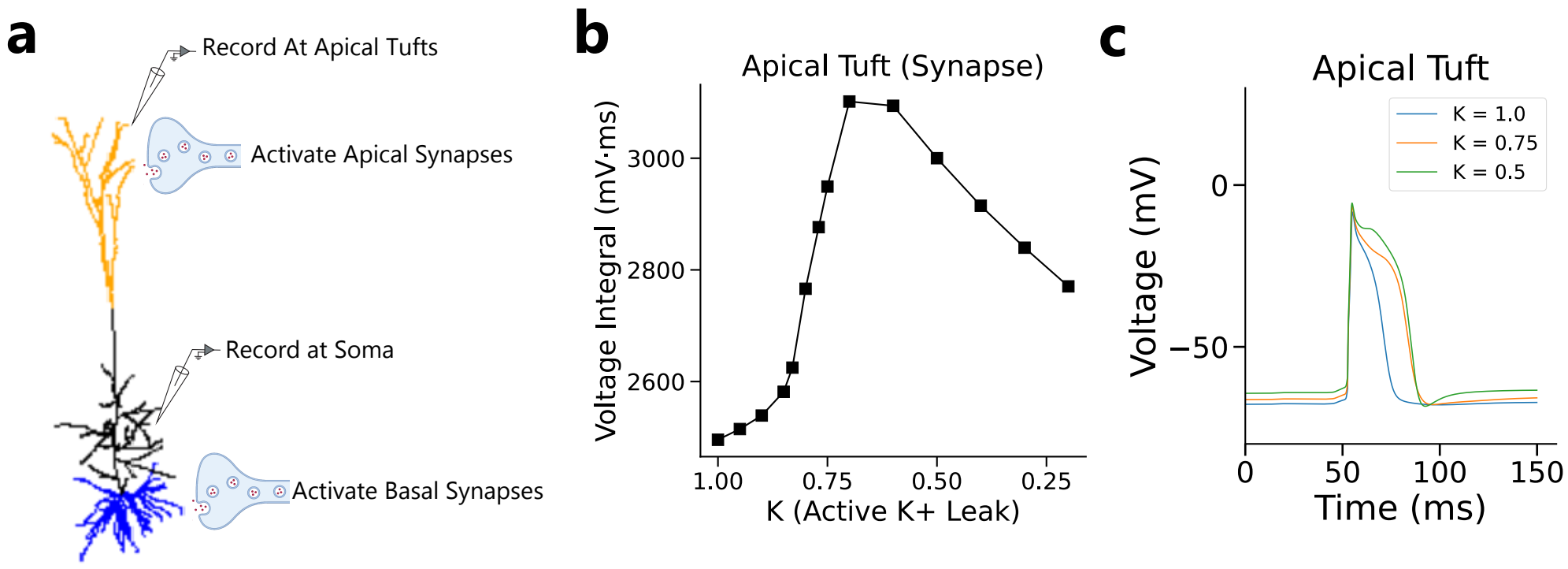


**Fig. S4 K2P suppression enhances dendritic NMDA plateau potentials (a.)** Experimental protocol in multicompartment model. A fixed coincident synaptic input is provided to synapses at apical and basal dendrites (11 tuft and 9 basal inputs) while simultaneously recording the membrane voltage at activated apical tufts and the soma. **(b.)** Voltage integrals changed non-linearly as $K$ decreased, revealing a sharp increase in the dendritic voltage integral with sufficiently small $K$ ($K < 0.8$) coinciding with the onset of a complete dendritic NMDA spike waveform. **(c.)** Example voltage traces at the apical tuft display an NMDA spike with sufficient mGluR-mediated suppression of K2P channels.

bioRxiv preprint doi: https://doi.org/10.64898/2026.07.25.740691; this version posted July 28, 2026. The copyright holder for this preprint (which was not certified by peer review) is the author/funder, who has granted bioRxiv a license to display the preprint in perpetuity. It is made available under a CC-BY 4.0 International license.

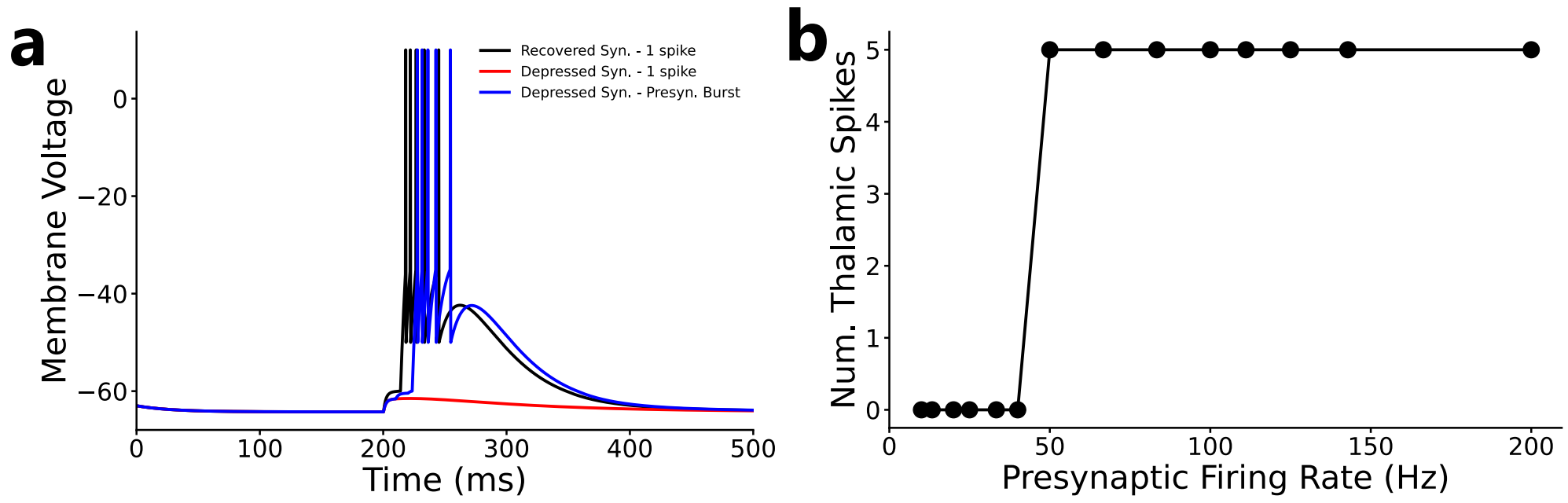


**Fig. S5 L5 Driver Synapses Requires High Presynaptic Firing Rates to Drive Thalamic Responses when synapses are Depressed (a.)** Simulated thalamic membrane voltage in response to L5 cortical input under three conditions: a fully recovered synapse receiving a single spike (black), a depressed synapse receiving a single spike (red), and a depressed synapse receiving a burst of 3 spikes at 100 Hz (blue). While a single input through a recovered synapse is sufficient to evoke a thalamic action potential, synaptic depression causes depolarization resulting from single spikes to be subthreshold. Only when presynaptic spikes arrive in rapid succession does the depressed synapse reliably drive thalamic firing. **(b.)** Number of thalamic spikes evoked as a function of presynaptic firing rate, for a fixed number of pulses (3 presynaptic events). Thalamic spiking is absent at low firing rates and emerges sharply only above a critical rate threshold, illustrating that high-frequency cortical activity is necessary to overcome synaptic depression and reliably recruit thalamic responses in L5 driver synapses.

bioRxiv preprint doi: https://doi.org/10.64898/2026.07.25.740691; this version posted July 28, 2026. The copyright holder for this preprint (which was not certified by peer review) is the author/funder, who has granted bioRxiv a license to display the preprint in perpetuity. It is made available under a CC-BY 4.0 International license.

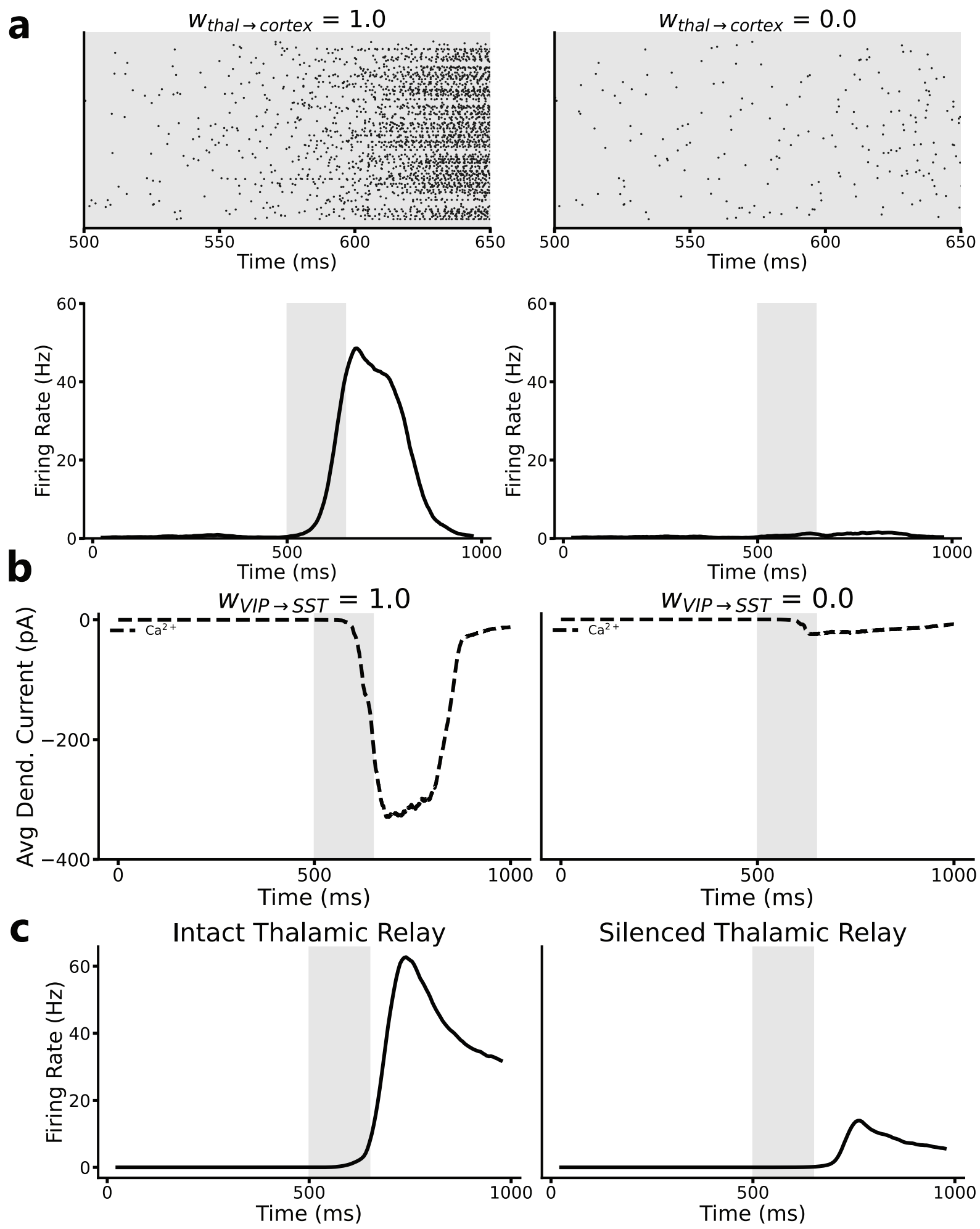


**Fig. S6 Network Design Constraints.** (**a.**) Raster plots of sensory-area neurons (top) and their population firing rates (bottom) with TC feedback onto sensory cortex intact ($w_{\text{thal}\to\text{cortex}} = 1.0$) versus removed ($w_{\text{thal}\to\text{cortex}} = 0.0$). Without thalamic feedback, stimulus-driven cortical activity is substantially reduced, consistent with [52]. (**b.**) Average dendritic $Ca^{2+}$ currents in sensory-area PNs with the VIP→SST disinhibitory motif intact ($w_{\text{VIP}\to\text{SST}} = 1.0$) versus removed ($w_{\text{VIP}\to\text{SST}} = 0.0$). Recruited by thalamic feedback via VIP interneurons, this motif gates dendritic $Ca^{2+}$ plateau potentials by opening temporally precise windows of reduced SST-mediated dendritic inhibition [53]. (**c.**) Decision-area population firing rate with the higher-order sensory thalamic relay to the decision area intact (left) versus silenced (right); silencing the relay markedly reduces the stimulus-evoked decision-area response with the network relying solely on cortico-cortical connectivity. Gray bars mark stimulus onset. Rasters show a single representative trial; firing-rate and dendritic-current traces are averaged over 10 trials of medium-strength stimulus presentation.

bioRxiv preprint doi: https://doi.org/10.64898/2026.07.25.740691; this version posted July 28, 2026. The copyright holder for this preprint (which was not certified by peer review) is the author/funder, who has granted bioRxiv a license to display the preprint in perpetuity. It is made available under a CC-BY 4.0 International license.

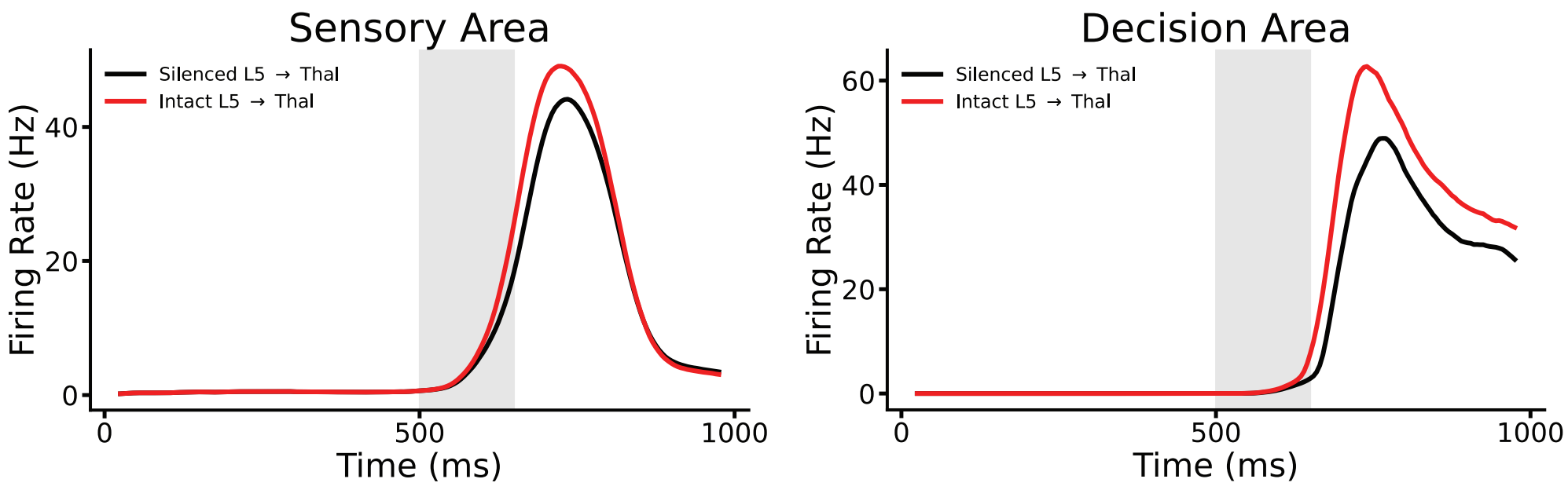


**Fig. S7 Silencing L5 to HOs thalamus projection attenuates perceptual detection.** Silencing L5 to HOs thalamus projections during stimulus presentation (black) reduces stimulus-evoked firing in both cortical areas. In the sensory area (left), this reflects the loss of depolarizing L5 CT input, which weakens TC modulation of sensory cortex. In the decision area (right), firing is further reduced because silencing this projection also removes the thalamic relay that links the two cortical regions, lowering effective connectivity between them.

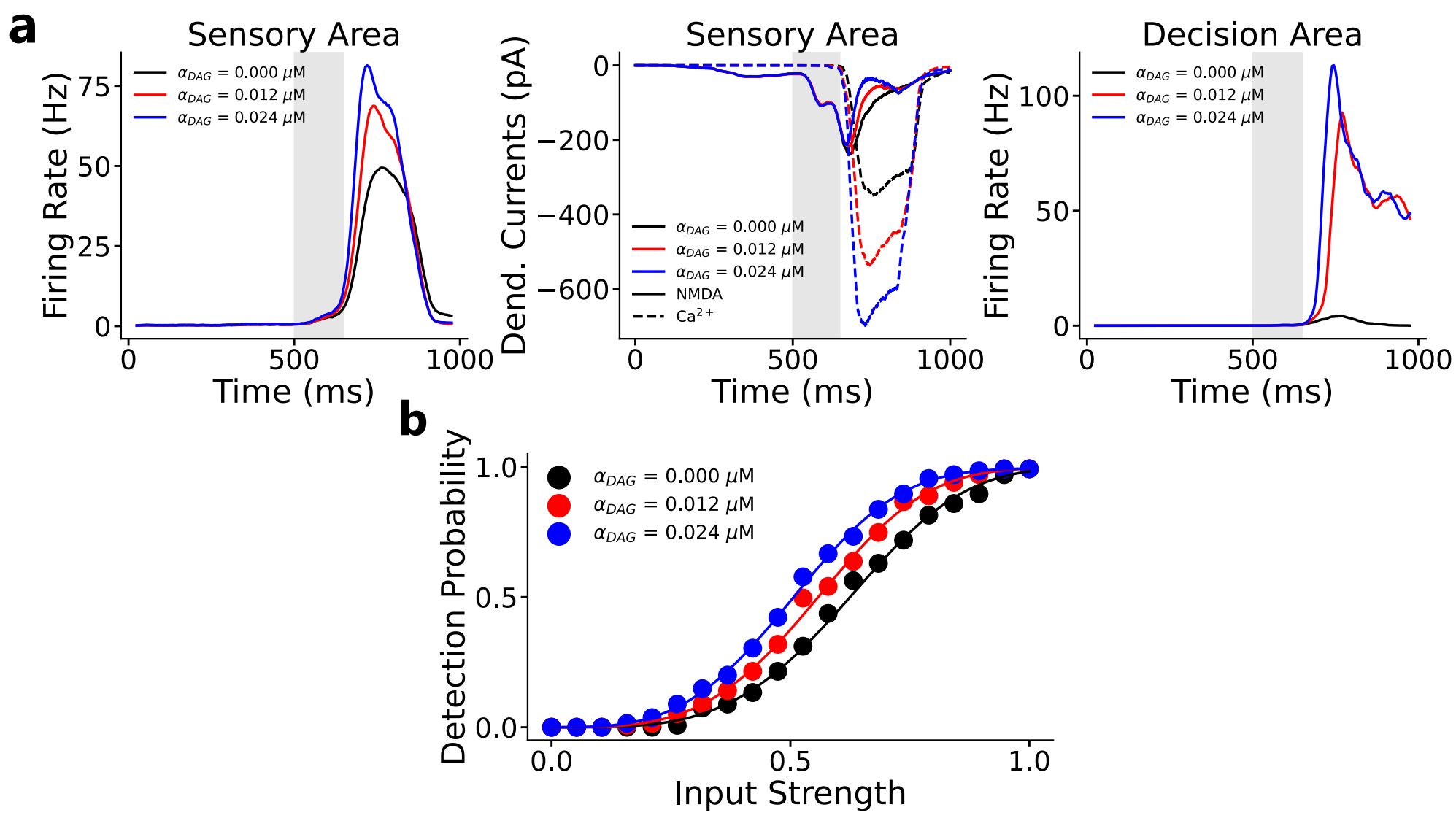


**Fig. S8 TC mGluR Activation Lowers Perceptual Threshold in the Tonic Firing Regime** We repeated the simulations of Fig. 4 with a 30 pA constant current injection to HOs thalamic neurons (in addition to their baseline CT mGluR depolarization), causing them to fire tonically rather than in burst mode. **(a.)** An example trial showing sensory area firing rates (left), sensory area dendritic currents (middle), and decision area firing rates (right) when we increase mGluR activation ($\alpha_{\mathrm{DAG}}$ = 0.000, 0.012, 0.024 $\mu$M). As was the case in Fig. 4, stronger mGluR engagement increases the firing of sensory neurons, reflected in larger voltage-dependent $Ca^{2+}$ (L-type) currents, and drives the decision area toward sustained activity. **(b.)** Detection probability as a function of input strength for the three levels of mGluR activation. Increasing $\alpha_{\mathrm{DAG}}$ shifts the psychometric curve leftward, lowering the perceptual threshold.

bioRxiv preprint doi: https://doi.org/10.64898/2026.07.25.740691; this version posted July 28, 2026. The copyright holder for this preprint (which was not certified by peer review) is the author/funder, who has granted bioRxiv a license to display the preprint in perpetuity. It is made available under a CC-BY 4.0 International license.

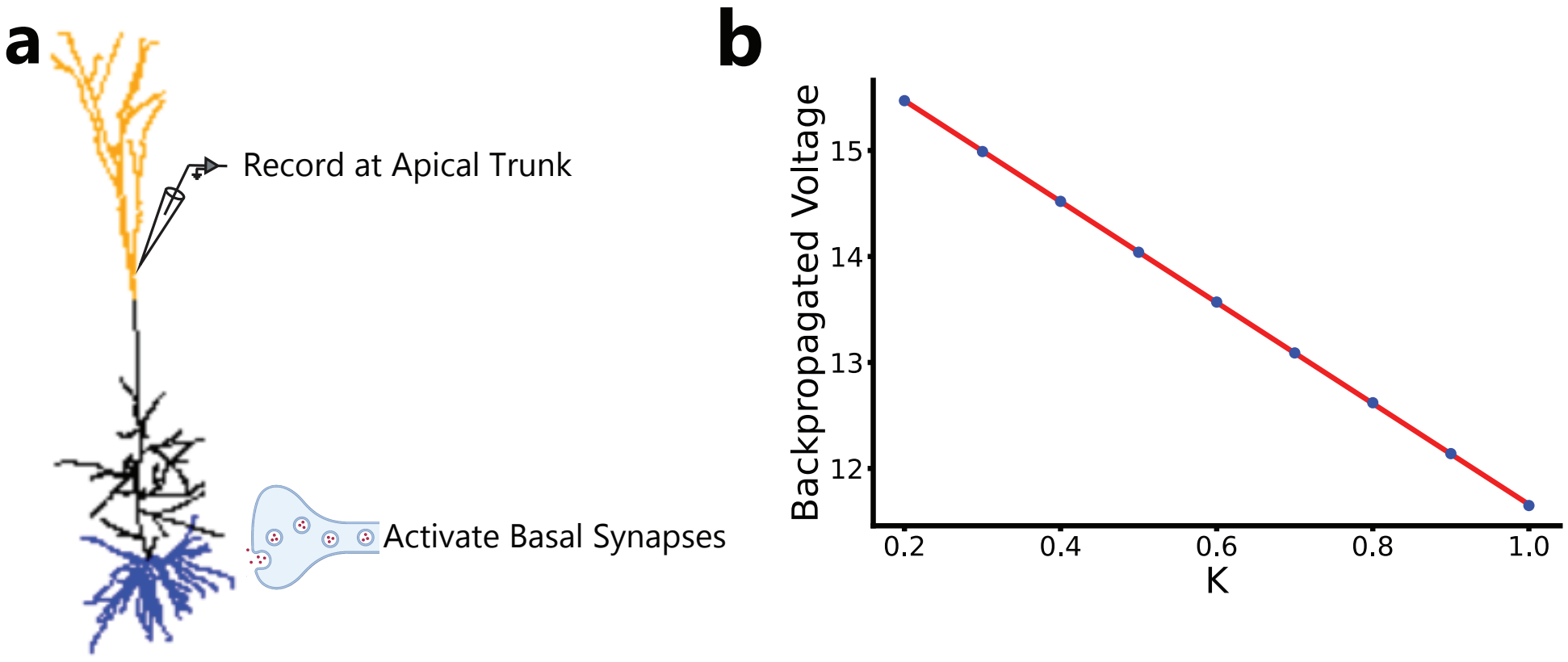


**Fig. S9** **Backpropagating action potential calibration** (**a.**) Schematic of the multicompartmental PN model used to calibrate the bAP in two-compartment PNs in the spiking network. Basal synapses are activated to elicit a somatic spike, and the resulting backpropagated voltage is recorded at the apical trunk. (**b.**) Relationship between the fraction of open K2P channels ($K$) and bAP amplitude measured at the apical trunk. A linear fit ($(16.431 - 4.783K)$ mV, red) captures the $K$-dependent voltage response used to set the bAP magnitude in the reduced two-compartment model.
